# Applying Implementation Science to Advance Electronic Health Record–Driven Learning Health Systems: Case Studies, Challenges, and Recommendations

**DOI:** 10.2196/55472

**Published:** 2024-10-07

**Authors:** Katy E Trinkley, Anna M Maw, Cristina Huebner Torres, Amy G Huebschmann, Russell E Glasgow

**Affiliations:** 1 Department of Family Medicine School of Medicine University of Colorado Anschutz Medical Campus Aurora, CO United States; 2 Adult and Child Center for Outcomes Research and Delivery Science Center University of Colorado Anschutz Medical Campus Aurora, CO United States; 3 Department of Biomedical Informatics School of Medicine University of Colorado Anschutz Medical Campus Aurora, CO United States; 4 Colorado Center for Personalized Medicine School of Medicine University of Colorado Anschutz Medical Campus Aurora, CO United States; 5 Division of Hospital Medicine School of Medicine University of Colorado Anschutz Medical Campus Aurora, CO United States; 6 Caring Health Center Springfield, MA United States; 7 Division of General Internal Medicine School of Medicine University of Colorado Anschutz Medical Campus Aurora, CO United States; 8 Ludeman Family Center for Women’s Health Research University of Colorado Anschutz Medical Campus Aurora, CO United States; 9 VA Eastern Colorado Geriatric Research Education and Clinical Center Aurora, CO United States

**Keywords:** learning health systems, implementation science, chronic care, electronic health record, evidence-based medicine, information technology, research and technology

## Abstract

With the widespread implementation of electronic health records (EHRs), there has been significant progress in developing learning health systems (LHSs) aimed at improving health and health care delivery through rapid and continuous knowledge generation and translation. To support LHSs in achieving these goals, implementation science (IS) and its frameworks are increasingly being leveraged to ensure that LHSs are feasible, rapid, iterative, reliable, reproducible, equitable, and sustainable. However, 6 key challenges limit the application of IS to EHR-driven LHSs: barriers to team science, limited IS experience, data and technology limitations, time and resource constraints, the appropriateness of certain IS approaches, and equity considerations. Using 3 case studies from diverse health settings and 1 IS framework, we illustrate these challenges faced by LHSs and offer solutions to overcome the bottlenecks in applying IS and utilizing EHRs, which often stymie LHS progress. We discuss the lessons learned and provide recommendations for future research and practice, including the need for more guidance on the practical application of IS methods and a renewed emphasis on generating and accessing inclusive data.

## Introduction

Nearly all health care settings in the United States use electronic health records (EHRs) to document the continuum of patient care [1,2], a trend that is increasingly evident in other countries as well [3-5]. Driven by federal incentives and regulations, health systems are being progressively encouraged or even mandated to document patient care in structured and actionable ways to facilitate reporting on quality of care metrics. As the breadth and depth of actionable data captured in EHRs expand, health systems are increasingly able to develop learning health systems (LHSs) [6,7]. In these systems, EHR data and other decision support features are utilized to advance the quintuple aim of health care: improving population health, promoting health equity, reducing health care costs, and enhancing both patient and care team experiences [8].

An LHS aims to “align science, informatics, incentives and culture for continuous improvement and innovation, with best practices seamlessly embedded in the health care delivery process in such a way that new knowledge is captured as an integral by-product” [9]. While the concept and the term LHSs are not new, significant progress has recently been made in their development, largely due to the growing capabilities of EHRs that make LHS more feasible. As interest in EHR-driven LHS increases, so does the interest in implementation science (IS), which plays a crucial role in ensuring that LHSs are feasible, rapid, iterative, valid, reliable, reproducible, equitable, and sustainable [10,11]. Both IS and LHSs aim to advance health and health care in ways that are locally relevant and externally valid, with IS providing methods and approaches that can help achieve these goals within LHS [10,12].

“IS is the study of how evidence-based practices are feasibly adopted, implemented, and sustained in real-world settings” [10,12]. A core aspect of IS is the use of its theories, models, and frameworks (TMFs), which are theory-driven approaches for evaluating the context in which LHSs are implemented and for guiding the selection of an implementation evaluation plan for a given LHS learning cycle project. IS and its TMFs draw on methods and theories from various disciplines and evolve over time as they are applied to new perspectives and situations, including different LHSs. A unique strength of IS and its TMFs is their capacity to adapt as new scientific methods emerge, while rigorously translating evidence into practice in ways that are locally relevant, yet generalizable, replicable, sustainable, and equitable [12]. Central to achieving the benefits of IS is the use of its TMFs, which offer a systematic and replicable approach to adapting evidence-based practices to local settings in ways that are both pragmatic and scientifically robust. IS TMFs can be integrated with broader LHS frameworks [13,14] to provide more specific guidance on aligning and evaluating the overarching LHS or learning cycle with the local context in ways that remain generalizable [10]. The adaptability of IS, along with its dual emphasis on locally relevant and externally valid findings, makes it particularly well-suited to support the goals of LHSs in their rapidly changing and inherently complex settings. While the success of LHSs undeniably requires a multidisciplinary approach, with theories and models from other disciplines playing a key role [15], this paper specifically highlights the value of applying IS to advance LHSs [16].

With the increasing use of IS TMFs for LHSs, certain inherent challenges have emerged, partly due to the limitations of EHRs [17,18]. Although EHRs serve as a rich data source, they often lack crucial data needed to effectively apply TMFs, such as key patient-reported outcomes and social determinants of health [19]. Such data are often too complex to collect consistently for most patients but are essential for informing patient-centered care decisions and evaluating the impact of a learning cycle. While EHR constraints challenge the application of IS TMFs, these frameworks can also help address some limitations of EHRs, such as expanding the use of EHR data beyond patient care and billing [19,20]. Nonetheless, there is a lack of guidance and ambiguities in applying IS TMF constructs to learning cycle projects, particularly when using EHR data. Additionally, some perceive TMFs as overly academic, with concerns that operationalizing them is resource-intensive and not practical, intuitive, or sufficiently rapid [18,21].

To advance the goals of LHSs, there is a critical need for guidance and support on pragmatically adapting and applying IS TMFs. This adaptation must align with the varying resources and expectations of different health systems and work within existing constraints, such as competing priorities, resource limitations, and data availability. The purposes of this paper are to (1) discuss the benefits and challenges of applying IS TMFs to EHR-driven LHS learning cycles; (2) outline the key features of a widely used IS TMF, the Practical, Robust Implementation and Sustainability Model (PRISM), as applied to LHS research; (3) provide 3 pragmatic case studies of this application; and (4) explore future directions, challenges, and opportunities for incorporating IS TMFs to support rapid LHSs and address the limitations of EHR data. We offer recommendations and resources for leaders, clinicians, implementers, quality improvement specialists, and researchers involved in developing an LHS or conducting learning cycles. These recommendations focus on how to feasibly adapt IS approaches and methods to various LHS situations.

## Methods

### Overview of PRISM Applications

In this article, we review 3 case studies illustrating the pragmatic application of an IS TMF and its associated methods, the PRISM, to LHSs. These examples highlight useful and practical applications of PRISM and serve as a basis for discussing challenges and future directions related to the use of IS methods and EHR data.

Although many IS TMFs exist [22,23], we selected PRISM to concretely illustrate the application of a TMF within an LHS. PRISM is an expanded version of the RE-AIM (Reach, Effectiveness, Adoption, Implementation, and Maintenance) framework, incorporating 4 context domains and the 5 RE-AIM outcome dimensions [17,24-27], as detailed in Tables 1 and 2. PRISM evolved from a blend of frameworks from agriculture (Diffusion of Innovation), engineering (Plan-Do-Study-Act quality improvement cycles), and health care (Chronic Care Model) [24]. PRISM is designed to be used throughout the life cycle of a study or project to guide the systematic assessment and alignment of the project with its context, aiming to maximize equitable impact on relevant outcomes and sustainability. PRISM underscores the importance of aligning a project with the diverse perspectives and characteristics of various partners, including those who will be directly or indirectly impacted or involved in approving and funding the programs, such as frontline staff, clinicians, clinic or department leaders, system-level leaders, and boards of directors. For effective alignment, it is crucial to ensure the representation of these partners. A key aspect of promoting equity is defining relevant outcomes that are important from diverse perspectives and measuring the representativeness of these outcomes across various demographics (patients) and types of clinics or providers (settings) [27]. PRISM’s RE-AIM outcomes facilitate discussions about relevant and meaningful outcome measures at different levels of perspective (eg, leadership, clinician, patient) while emphasizing representativeness and pragmatic issues such as adoption and uptake. PRISM also takes into account the external context (eg, policies, guidelines) and the support or infrastructure available for initial implementation and sustainability (eg, resources, audit, and feedback processes). This consideration enhances the likelihood that the project will continue beyond the study timeline or funding. The systematic approach provided by a TMF like PRISM, combined with the RE-AIM pragmatic outcomes, allows a project to be adapted and scaled up in ways that are locally relevant to different situations and health systems.

To illustrate the challenges and potential solutions of applying IS TMFs to LHS learning cycles and using EHR data, we present retrospective case studies of 3 applications of PRISM to different LHS projects across diverse health care settings. These case studies were selected based on the authors’ experiences to represent various types of projects and settings, as well as different approaches to applying PRISM and addressing key issues within LHSs. We focus on identifying challenges and solutions related to (1) operationalizing and applying PRISM within the EHR-embedded LHS context; (2) pragmatically adapting PRISM and its RE-AIM outcomes based on available resources, expectations regarding speed, and data availability; and (3) effectively utilizing EHR data. The solutions provided aim to offer guidance and direction on addressing these challenges and improving the practical application of PRISM and other TMFs and IS methods to EHR-driven LHS.

**Figure 1:**
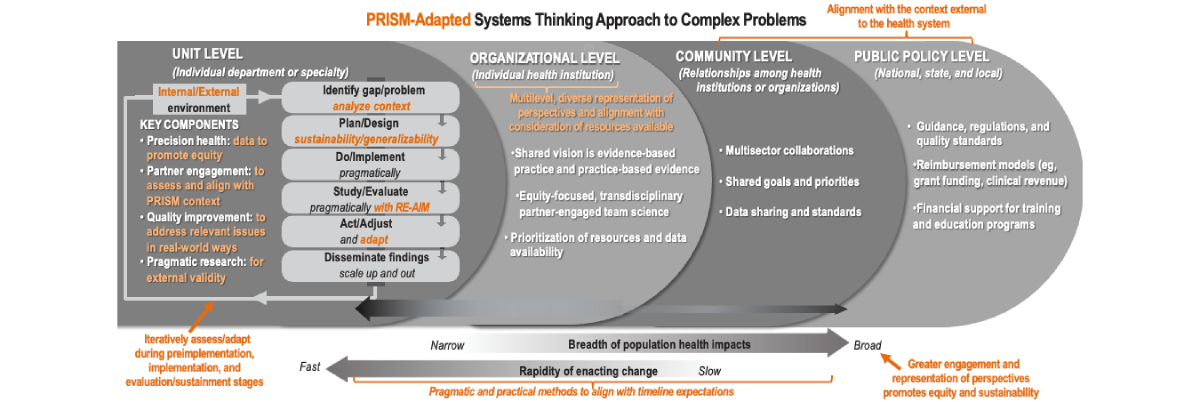
PRISM-adapted conceptual model of a fully mature learning health system NOTES: The orange denotes ways PRISM helps guide and inform key components and activities of a fully mature LHS, including: alignment of a project with the context internal and external to the health system to ensure local relevance and external validity; consideration of inclusive precision health data to promote equity; engagement and integration of diverse partner perspectives to assess and align a project with the context in ways that promote equity and sustainability; iterative assessments and adaptations across all stages of a project (pre-implementation planning, implementation and evaluation/sustainment) to increase speed, effectiveness, and sustainability; use of pragmatic RE-AIM outcomes that consider equity and issues important to partners; and adapting how PRISM is applied based on available timelines and resources.

To facilitate a systematic evaluation of how PRISM was applied in each case study and to minimize recall bias, we first adapted our previously published framework for a fully mature LHS to illustrate where and how PRISM and its associated methods can be applied (Figure 1) [10]. The original framework was designed to be agnostic to any specific IS TMF, so we modified it by overlaying PRISM and highlighting where and how it integrates with the broader LHS framework. In this figure, PRISM guides and informs key aspects and activities of an LHS, including representativeness and equity of perspectives and outcomes; achieving local relevance and external validity or generalizability; rapidity of change and impact; and designing for sustainability. This adapted figure of a fully mature LHS was used to stimulate recall and identify challenges and solutions when applying PRISM and using EHR data. For each case study, we thematically reflected on the challenges and both actual and potential solutions at each phase of the implementation continuum (ie, preimplementation or planning, implementation, and sustainment or evaluation) [28]. This reflection was conducted both inductively to identify new themes and deductively by considering preidentified themes. We also identified aspects of the EHR that pose barriers to applying PRISM and other IS TMFs, which are crucial to address in order to achieve the aspirational goals of a high-functioning LHS.

**Table 1:** PRISM’s^a^ contextual domains.

PRISM context domain	Description
Patient and organizational *characteristics*	The characteristics, priorities, and needs of the setting, including those affected by or involved in the intervention, are crucial to consider when designing the intervention. LHSs^b^ should design learning cycles to align with the priorities and values of the setting.
Patient and organizational *perspectives* of the intervention	The setting’s view of the intervention, including the perspectives of those directly and indirectly affected, influences its uptake and impact. LHSs should prioritize learning cycles that address local gaps in ways that are both relevant and acceptable to the setting.
Implementation and sustainability infrastructure	The time, staff, and money required to feasibly implement and maintain the intervention are crucial considerations. This also includes alignment with existing processes, norms, and priorities to promote sustainability. LHSs must consider the available resources and supporting infrastructure to ensure sustainability and design learning cycles with lasting effects.
External environment	This includes clinical practice guidelines, policies or regulations, reimbursement mechanisms, and other incentives such as national benchmarking. LHSs are influenced by contextual factors outside their health setting and consider published literature when prioritizing and designing learning cycles.

^a^PRISM: Practical, Robust Implementation and Sustainability Model.

^b^LHS: learning health system.

**Table 2:** PRISM’s^a^ RE-AIM^b^ outcome dimensions.

RE-AIM outcome dimension^c^	Description
Reach	Who was intended to benefit and who participated or was exposed to the intervention? Representativeness and equity of reach LHSs^d^ should assess what proportion of the target group was impacted and consider the characteristics of those affected to ensure representativeness and equity in reach.
Effectiveness	What was the most important benefit you were trying to achieve and what were the negative outcomes (eg, safety issues)? Include quality of life and equity of outcomes. LHS should evaluate the impact from various perspectives and compare the characteristics of those who were positively and negatively affected.
Adoption	Where was the intervention applied and who applied it, and who declined? LHSs should examine how and why uptake or use varies among different users and settings.
Implementation	How consistently was the intervention delivered? (Fidelity) How was it adapted? How much did it cost? Equity and representativeness (subgroup effects) across implementation outcomes LHSs should consider how and why adaptations are made based on different contextual influences, as well as the costs associated with initial and ongoing implementation. This information is crucial for making informed decisions about local sustainability and scalability.
Maintenance	How long was the intervention sustained and how long are the results sustained? Equity and representativeness of maintenance LHSs should plan for and assess sustainability and contextual drivers

^a^PRISM: Practical, Robust Implementation and Sustainability Model.

^b^RE-AIM: Reach, Effectiveness, Adoption, Implementation, and Maintenance.

^c^Issues of proportion and representativeness of participants compared with all those eligible or those who decline are important and relevant across all RE-AIM dimensions.

^d^LHS: learning health system.

### Case Studies

#### Overview

Below, we provide a brief description of each LHS-based project where PRISM was applied. Table 3 offers an overview of the different features of these case studies.

**Table 3:** Overview of the key differentiating features of the case studies.

Feature	EHR^a^-embedded CDS^b^ for heart failure [29]	EHR-based dashboard for LUS^c^ [30]	FQHC^d^-led social needs screening and response equity study
Problem addressed	Gaps in guideline-concordant prescribing of beta-blocker medications for patients with heart failure	Address the heightened need for accurate bedside chest imaging during the COVID-19 pandemic by implementing lung ultrasound among hospitalists.	Define a multipartner implementation plan for social risk screening and response equity to address the CMS^e^ mandate and emerging Medicaid ACO^f^ equity scores.
Setting	The EHR of 28 primary care clinics in a large integrated health care system	A quaternary care academic medical center	Partnership of multiple FQHCs
Target audience	Primary care clinicians	Hospitalists	Researchers and health center/ACO leaders and staff
Intervention and implementation strategies	A CDS tool was integrated into clinical EHR workflows to alert clinicians and recommend initiating a beta-blocker medication during patient visits.	Hospitalist training in lung ultrasound and the use of iterative PRISM^g^ to implement lung ultrasound in the management of patients with COVID-19.	Collaboration across FQHCs to develop a plan using the iPRISM^h^ webtool iteratively as a strategy to examine social risk screening and response equity in IS^i^ research.
How PRISM was used	For the planning and sustainment/evaluation phases	Iteratively in the planning and implementation phases	Iteratively in the planning phase for cocreation

^a^EHR: electronic health record.

^b^CDS: clinical decision support.

^c^LUS: lung ultrasound.

^d^FQHC: federally qualified health center.

^e^CMS: Centers for Medicare and Medicaid Services.

^f^ACO: accountable care organization.

^g^PRISM: Practical, Robust Implementation and Sustainability Model.

^h^iPRISM: Iterative Practical, Robust Implementation and Sustainability Model.

^i^IS: implementation Science.

##### Case Study 1: EHR-Embedded Clinical Decision Support for Heart Failure

PRISM was used to design and evaluate a clinical decision support (CDS) tool that recommended prescribing a beta-blocker for primary care clinicians treating patients with heart failure with reduced ejection fraction [29,31]. PRISM guided the systematic assessment of context, which included the following:

- focus groups with patients to understand their treatment preferences and needs;
- iterative, multilevel user-centered design and testing procedures, including focus groups and semistructured interviews with clinicians and clinician leaders; and
- meetings with executive-level informatics and operational leaders, and governance groups.

Based on this iterative partner engagement process, the CDS tool was designed to align with the context while also addressing the technical and data limitations of the EHR. The process also involved identifying pragmatic RE-AIM outcomes that were important and relevant from various perspectives.

A total of 28 primary care clinics were cluster-randomized to either the new, contextually customized CDS tool or an active control group for 6 months. PRISM was used to guide a mixed methods evaluation and to identify adaptations that would promote sustainability and expand the scale of the CDS tool. Specifically, adoption and effectiveness were quantitatively assessed using EHR data. Clinicians were interviewed to determine whether either of the CDS tools should be continued and to understand what changes were needed to optimize adoption and effectiveness within their workflows. Based on the evaluation, the customized CDS tool was found to be more effective, and clinicians expressed a preference for its continued use. As a result, all clinics have since transitioned to the customized CDS tool, and its effectiveness has been sustained [32]. It was also determined that the CDS tool needs to include additional evidence-based medications for heart failure and should be expanded to cardiology clinics. Plans are underway to broaden the tool’s scope to include these medications and extend its use to cardiology clinics.

##### Case Study 2: EHR-Based Dashboard for Lung Ultrasound

In this pilot implementation study, an iterative assessment process was used to ensure high-fidelity and equitable implementation of lung ultrasound for patients hospitalized with COVID-19 [30]. This was achieved through a recurring mixed methods evaluation of prioritized PRISM constructs, including context and progress on outcomes. The evaluation incorporated qualitative interviews and an operational dashboard that displayed prioritized RE-AIM outcomes using real-time EHR data.

Specifically, PRISM was used to assess context by guiding the interview questions posed to multilevel partners during the planning and implementation phases of this study. The questions were designed to identify and characterize barriers to implementation and to uncover potential strategies for overcoming these barriers. The contextual data collected were then used by implementers to inform iterative adaptations to implementation strategies, with the goal of improving PRISM’s RE-AIM outcomes. Additionally, prioritized outcomes were iteratively assessed quantitatively using an *operational dashboard* that displayed the representativeness and extent of reach and adoption of lung ultrasound.

The implementation team met every 2 weeks to review outcomes displayed on the dashboard and the qualitative interview data. They assessed barriers to progress in reach and adoption and screened for evidence of emerging disparities in implementation. Based on these assessments, the team collaboratively decided which implementation strategies to deploy, adapt, or discontinue. *This iterative PRISM approach,* combined with an operational dashboard, offered a low-burden method for monitoring progress and disparities in implementation, as well as for making timely, data-driven adjustments to implementation strategies. This approach has since been expanded to support the equitable implementation of additional evidence-based applications of lung ultrasound (eg, management of heart failure) and other point-of-care ultrasound applications within the same health system.

##### Case Study 3: Social Needs Data Within Federally Qualified Health Centers

In this ongoing project, PRISM is being used to support cross-institutional partnership engagement and the cocreation of an LHS project focused on social risk screening and response equity across multiple federally qualified health centers (FQHCs). Building on long-standing collaborative research by an FQHC in Springfield, Massachusetts, the project brings together partners from a model B Medicaid Accountable Care Organization comprising multiple FQHCs in Massachusetts, collaborators from the Massachusetts Primary Care Association, and research partners from Harvard’s Implementation Science Center for Cancer Control Equity. The focus of the project was on mandated universal social risk screening and response requirements for FQHCs [33,34]. The team used PRISM to assess each partner’s perspective on anticipated RE-AIM outcomes and to identify contextual issues of importance.

To operationalize PRISM, the team used the Iterative Practical, Robust Implementation and Sustainability Model (iPRISM) webtool [17] during the preimplementation planning phase. The iPRISM webtool includes 21 assessment questions designed to systematically guide individuals or teams through the process of assessing context and anticipated RE-AIM outcomes for projects, while also facilitating shared input or cocreation. The iPRISM webtool was developed to help implementers efficiently apply PRISM to various types of projects and support implementation teams from diverse backgrounds (eg, clinicians/researchers with and without IS expertise). Each partner completed the iPRISM webtool independently (n=6; responses from 1 partner were not linked because they selected a different stage/phase within the webtool form).

During 2 sequential debrief meetings, the partners reviewed their responses, which led to increased clarity on (1) the RE-AIM and PRISM factors with lower scores, highlighting areas for priority focus; and (2) the variation in scoring across different partner perspectives, which contributed to a better understanding of each FQHC and defined multilevel assessment opportunities. Examining and discussing the mean scores and ranges of responses for each item provided valuable insights into both aspects. The tangible outcomes from this process included the development of a cocreated set of specific aims for the project, a better understanding of each FQHC’s perspective and contextual issues, and consensus among the partners to apply for grant funding to support the ongoing work as an innovation in FQHC-led social care research.

## Results

### Challenges, Solutions, and Future Directions

In each case study, we identified several challenges, potential solutions, and considerations for future research when using EHR data, PRISM, and other IS approaches. The detailed evaluation and findings from each case study are described in Multimedia Appendix 1 and are categorized into 6 overarching themes of challenges with corresponding solutions. These themes and solutions are described below and summarized in Table 4. Across these themes, there are interdependencies, which are expected from a systems science perspective. For instance, resource and time constraints can exacerbate EHR data limitations, and varying levels of IS expertise can complicate the appropriate application or adaptation of PRISM and IS methods. For each challenge, we discuss the issues,potential solutions,and future directions.

**Table 4:** Challenges, solutions, and future directions.

Theme of challenge	Description of challenge	Potential solutions or ways to mitigate challenges and future directions
Team science issues: *LHS*^a^ *and IS*^b^ *involve diverse teams with different backgrounds and perspectives*	Level setting of vocabulary and terminology that is new or has different meanings in different fields Shared goals and understanding of the problem and project are important Can be difficult to moderate and understand different perspectives and ensure openness in sharing different perspectives Consistency in applying TMF^c^ to assess context/outcomes across individuals of a team	Employ team science best practices [35], including the creation of a shared vision and understanding of each partner’s perspectives. Use the iPRISM^d^ webtool to facilitate the use of team science best practices; level set vocabulary, issues, and goals; identify and summarize different perspectives; and consistently apply a TMF across partners.
Limited/no IS experience: *LHS teams may have limited IS expertise*	Makes the application of IS difficult which is a barrier to using these methods that aim to improve relevance, sustainability, scalability, and equity	Identify external expertise or a consultant Leverage existing resources and tools that make IS more accessible (eg, iPRISM webtool; see Multimedia Appendix 2 for additional tools/resources) Create additional resources to increase the accessibility of IS, including guidance on how to (1) feasibly and systematically anticipate, mitigate, and assess for unintended consequences including exacerbation of inequities, and (2) design for sustainability, including identifying and securing resources Invest in capacity building of IS including training and resources aimed at implementer/practitioner education in addition to implementation scientist training
Data and technology limitations: *LHS and IS methods are limited by the data available*	LHSs often rely on EHR^e^ data and the value of these data is limited by completeness, accuracy, equity, biases of documentation, or is difficult to capture because it is unstructured Contextual data are often not documented or accessible in structured formats, which often limits contextual assessments to qualitative analyses which can be limited by partial or small samples Reliance solely on quantitative or qualitative data limits a full picture of the context and impact or outcomes (eg, issues of actual vs stated and depth of understanding) Accessibility and functionality of software and technology to manage and use data can limit use	Proactively consider potential data issues and the implications for a given project, and develop workarounds (eg, proxies) or strategies (eg, transparency in reporting) to mitigate the negative impact Promote better/different data collection practices to ensure high-quality, unbiased, and inclusive data documented in standardized and structured ways Develop more guidance on how to qualitatively assess context when not accessible via quantitative data sources Engage diverse partners in the decision of what data are collected and how they are collected Invest in capacity building of personnel skilled in: making timely/relevant data accessible to implementers/system leaders (eg, can build dashboards) using advanced analytics such as natural language processing to transform data from unstructured to structured formats Invest in capacity building of software and technology that is more accessible and tailored to the needs of LHS including: transforming unstructured data into structured formats analyzing data and images intuitively conveying complex data in meaningful visualizations and other ways on demand using decision support and other tools that are more precise and able to nimbly embed within existing clinical and EHR workflows interoperability or integration across different EHRs and health systems
Time and resource constraints: *LHS and IS methods must fit within available timelines and resources*	Health system or other timelines may be fast and constrain LHS projects and IS methods System-level and implementation team–level resources and time may limit data access; collection of quality, representative, and iterative quantitative and qualitative data to assess context that dynamically changed over time; type, intensity, and frequency of partner engagement and other contextual assessment methodology that can be conducted; and sustained support. Evaluations of context and outcomes may be limited to what is discretely documented unless skilled staff is available to apply advanced analytic approaches (eg, natural language processing) to abstract unstructured data availability of skilled staff to query data and generate a report staff time to manually collect data The availability of skilled staff to pragmatically design and evaluate projects can limit internal or external validity	Avoid the trap of perfect and aim for a minimum viable product when there is no anticipated harm Collaborate to extend resource availability with: trainees who can contribute meaningfully while gaining valuable experience and knowledge methodologists who can provide needed skills while gaining authorship opportunities Apply IS “designing for sustainability” principles to plan for sustainability and develop the supporting infrastructure from the beginning Leverage advanced analytics (eg, natural language processing, machine learning) and data visualization platforms (eg, dashboards) to automate data collection, analysis, and reporting back to implementers and partners which can: make iterative assessments of context and outcomes feasible and sustainable increase the speed of positive impact or change when data are acted on improve overall efficiency Invest in capacity building of personnel with the skills that can build low-burden means (eg, dashboards) that allow for evaluation of process and effectiveness outcomes in real time
Appropriateness of certain IS principles, outcomes, and TMF constructs: *IS needs to adapt to each situation*	Some aspects may not be relevant or perceived as important Some aspects may not apply or some outcomes may not be addressed because of: resource or data constraints (eg, limit iterative assessments of context or adaptations, deter costing analyses) expectations around speed (eg, system priorities or patient safety issues require quick action) anticipated benefits or needs preclude resource allocation for certain evaluations (eg, cost, unintended consequences) difficulty measuring certain outcomes (eg, rare clinical outcomes or time to event, denominators unavailable to assess representativeness) or establishing reasonable causality (eg, unable to control for other influences) Application of IS can be challenging when the intervention is mandated and expected to change but is not evidence based and yet there is a need to (1) design for future and ongoing sustainability and (2) get buy-in from key partners evidence for an intervention varies by contextual situation (eg, all CDS^f^ interventions are not equal)	Create best practices and guidance on how/when to adapt IS principles, outcomes, and TMF constructs for diverse projects and situations Avoid the trap of perfect and work within the constraints of the available timeline, data, and other resources More guidance is needed on how to apply IS to interventions that are mandated without an established evidence base including how to: use an iterative LHS approach to evaluate effectiveness at intervals under different situations and inform intervention adaptations until it is evidence based (ie, effective) shift the incentive for partnership and buy-in from the strength of evidence to the requirement to implement use a “designing for sustainability” approach to assist in prioritizing resource allocation for a project that is likely to change as the evidence evolves For interventions with effectiveness that varies by contextual situation, mixed methods evaluations and transparent reporting of contextual factors will provide clarity of the conditions needed for effectiveness
Representation and equity: *LHS and IS need to proactively promote and assess for equity in perspectives and outcomes*	Partner engagement that does not represent the spectrum of perspectives: biases a project toward certain perspectives and priorities stymies equity Incomplete access to accurate data limits the ability to evaluate the equitable impact of projects assess for unintended consequences such as exacerbation of inequities	Aim to represent the perspectives of partners engaged at each stage of the project (planning, implementation, and evaluation), rather than just an average perspective across all stages. Use the iPRISM webtool [36] and other tools to systematically capture different perspectives Consider less traditional data sources and methods such as crowdsourcing and social media to expand the representation and inclusion of diverse data types Proactively consider potential inequities inherent within quantitative or qualitative data sources and any potential unintended consequences Transparently report perspectives engaged and completeness of data evaluated to guard against lack of diversity in perspectives that inform a project

^a^LHS: learning health system.

^b^IS: implementation science.

^c^TMF: theories, models, and frameworks.

^d^iPRISM: Iterative Practical, Robust Implementation and Sustainability Model.

^e^EHR: electronic health record.

^f^CDS: clinical decision support.

### Challenge 1: Team Science

Team science refers to the cross-disciplinary collaboration necessary to address scientific questions and challenges [37]. Both LHS and IS require multilevel engagement from partners with diverse roles, backgrounds, and perspectives, which introduces complexities. Representatives from various partner groups (eg, patients, community members, clinicians, leaders, nonclinical staff, and researchers from different fields) bring unique histories, perspectives, terminologies, biases, assumptions, and knowledge. Sometimes, communication issues or differences in perspectives are recognized, but they can often remain unnoticed for extended periods.

These team science challenges are manageable and should be addressed during the planning phase and throughout the project [35]. Taking the time to understand and respect each partner’s perspective and developing a shared vision and vocabulary is essential for team effectiveness and efficiency. It is also important to continually emphasize that different perspectives are not only beneficial but also expected [35]. The use of tools such as the iPRISM webtool [17,36] can systematically capture the diverse perspectives on teams and summarize the mean and distribution of scores, which can be used to focus team discussions on areas with lower mean scores (indicating areas for improvement) or where scores vary and perspectives differ. In the future, greater use and availability of tools such as the iPRISM webtool are recommended to facilitate team science principles within LHS and when using IS, including other TMFs [38,39] beyond PRISM.

In the social needs example, the diverse partner team included researchers and executive-level leaders with a range of IS expertise. The iPRISM webtool offered a grounded and shared entry point and opportunity to contextualize each partner’s perspective and expertise by providing (1) a guided process assessment, (2) a framework to understand the team’s similarities and differences, and (3) a shared language. This resulted in deepening the understanding of the range of potential themes related to social risk screening and response to be addressed and the various contexts in which they need to be considered.

### Challenge 2: Limited or No Implementation Science Experience

In an LHS, and within health systems more generally, there may be variable or no IS expertise, which can preclude the application of IS altogether or lead to inconsistent or incomplete implementation. Inconsistent application may result in replication issues or incomplete assessments of context or project alignment, ultimately affecting project outcomes and sustainability. Understanding how to apply IS principles and TMFs such as PRISM can be challenging without training, and it is often impractical to train all team members.

One potential solution is identifying external IS expertise, which may be feasible through existing consultation services [40-42]. Utilizing resources and tools that make IS more accessible to individuals and teams by simplifying its application can also help. Tools such as the iPRISM webtool [17] and other resources [43-50], which can increase the accessibility of IS, are described in Multimedia Appendix 2. In our case studies, we identified specific aspects of applying PRISM and IS generally that would benefit from greater guidance and tools, such as how to feasibly and systematically anticipate, assess for, and mitigate unintended consequences—including those that can exacerbate or create inequities—and how to design for sustainability, including identifying and securing resources. Capacity-building efforts are also needed to train implementation researchers and practitioners [51].

In the social needs example, the iPRISM webtool was used successfully to standardize the application of IS across a team that had variable IS experience, including some members who had no IS experience.

At the time of the heart failure and lung ultrasound examples, the lead researchers were being mentored through an institutional implementation science K12 program and are now independent implementation scientists supporting others’ LHS projects.

### Challenge 3: Data and Technology Limitations

Timely and feasible access to complete, accurate, and actionable quantitative and qualitative data is a universal challenge for LHS and IS [6,10,52-54]. These challenges limit outcome evaluations, contextual assessments, and the capacity to rapidly and strategically design interventions and programs that are equitable and optimally fit within workflows [55-58]. Often considered together under the umbrella of “informatics,” access to the needed technology is also a challenge. Even when technologies are accessible, they do not always have the functionality needed to seamlessly embed within clinical workflows. Furthermore, there may not be enough skilled staff to use or configure the technology to achieve the goals of delivering the “right” information at the “right” time, to the “right” person, in the “right” format, and through the “right” channel [59].

When faced with data and technology limitations, it is important to first recognize the potential issues and how they might impact a project, and then explore workarounds or strategies to monitor for potential downstream consequences. Workarounds to address data access issues include creating proxies or estimates and conducting sensitivity or subgroup analyses, which may not be precise but can provide valuable insights and aid in understanding. Addressing technology functional limitations often requires adopting a “good enough” mindset, provided the benefits are expected to outweigh potential sacrifices in user experience and no harm is anticipated [60,61]. Other strategies include transparently reporting the data and acknowledging technology limitations, allowing the audience to make informed interpretations of the findings. The current reality may compel LHS projects to operate within existing constraints, but it is crucial to advocate for the goals of truly inclusive precision health [62], which requires comprehensive integration of data to support holistic care decisions. Guidance is needed to help current LHS teams optimize their projects and evaluations within these constraints. However, it is also important to challenge and push against current limitations when necessary. For example, when available data are known to produce biased information or fail to include critically important patient or contextual factors, the health system may need to add essential data elements or utilize novel methods [63,64] to achieve impactful and equitable results [56,62,65,66].

In the lung ultrasound example, reach was trended month to month as a prioritized outcome to monitor the progress of implementation. However, the reach presented as simply the percentage of eligible patients that received lung ultrasounds was somewhat misleading because the denominator, the number of patients hospitalized with COVID-19, fluctuated so greatly with each surge of the pandemic. In this case, it seemed more transparent and less misleading to present reach with the absolute value of the numerator and denominator visible as opposed to just a percentage. The heart failure example illustrates ways to work within the functional constraints of technology that is good enough and proactively considers potential unintended consequences.

### Challenge 4: Time and Resource Constraints

In most projects, restrictions on time, availability of skilled personnel, and other resources can slow data access or preclude it entirely. Additionally, the time required from the implementation team and participants can limit the breadth of perspectives engaged and the frequency of qualitative assessments. These limitations can negatively impact the equity and sustainability of projects.

To make progress, LHS teams often need to adapt evaluation plans and partner engagement methods to fit within available time and resource constraints. Finding partners and collaborators who can generate win-win situations and extend resources is also crucial for overcoming these limitations. There is also a growing availability of consultant-type services to support LHS initiatives, offer specific methodological expertise, or connect projects with needed resources [67,68]. Advances in artificial intelligence, such as natural language processing and machine learning, can enhance efficiency and reduce resource usage by automating data collection and analysis. Although these advancements are increasingly present within LHS, improving accessibility to these skills, resources, and software for automation is essential for further enhancing efficiency. Additionally, while the application of artificial intelligence approaches can offer significant benefits, it is important to exercise caution and carefully balance and vet automated processes [69,70]. To further improve efficiency, creating and disseminating “how-to” or implementation guides and recommendations [42,71-73] could help reduce the resources needed to understand, adapt, or apply specific technologies, data, or methodologies.

In the lung ultrasound example, due to the limited time available to the implementers between iterative PRISM cycles, the qualitative interview data collected were not as systematically analyzed as is ideal. In the social needs example, a partner was not able to engage in all iPRISM webtool planning and contextual assessment activities due to time constraints, thus measures were taken to ensure they were able to engage via asynchronous methods, provide summary updates on progress, and gain full team consensus at various points in the process. In the heart failure example, resource availability prevented the ability to iteratively assess for and make adaptations, which may have led to more impactful outcomes.

### Challenge 5: Appropriateness of IS Principles, Outcomes, and TMF Constructs

Not all aspects of IS apply to every LHS situation for a variety of reasons. In some cases, a TMF construct or IS method may not align with a project’s goals, may not fit within resource or timeline constraints, or may need to be adapted to suit the situation [74]. Additionally, when implementing interventions without an established evidence base, flexibility is required in applying IS methods and partner engagement strategies. For instance, the LHS may be implementing a clinical guideline recommendation based on poor-quality or low-strength evidence, or following a new regulatory mandate for an intervention that has yet to demonstrate effectiveness [75].

IS is intended to be practical and pragmatic, and its principles, outcomes, and TMF constructs should be considered as a guide and adapted to the situation at hand, focusing on what is feasible and relevant for the context [74]. When changes are made, it is important to document and report the adaptations along with the rationale to facilitate future scalability. For projects without an established evidence base, some common IS strategies, including how and which partners are engaged, may need to be adjusted. Further, when a project’s evidence base is uncertain or may vary based on contextual conditions (eg, the effectiveness of a CDS alert varying by clinical situation and design), iterative IS and LHS approaches can be leveraged to develop the evidence base and understand the necessary conditions for success.

To increase the uptake of IS, it is essential to promote awareness that IS should be adapted to fit the specific needs and context of each project [76,77]. Current misconceptions that IS cannot be adapted may inhibit its application. Providing guidance on how to adapt TMFs and IS methods, supported by case examples, can help address these misconceptions and enhance the accessibility and use of IS. Additionally, interactive tools such as the iPRISM webtool, which dynamically guides implementers through the process of adapting IS methods for their specific project, could further facilitate this adaptation.

In the heart failure example, the adoption measure was modified from the original definition to be relevant to the situation at hand and to still facilitate the collection of important implementation outcomes.

In the social needs example, the intervention was not yet evidence based, which changed aspects of IS partner engagement. Specifically, partner buy-in shifted from a shared understanding of the effectiveness to a shared incentive to meet the mandate with a common interest in contributing to the development of an evidence base. Across all 3 examples, none were able to assess all of PRISM’s RE-AIM outcomes or evaluate the cost of implementation due to data and resource constraints as well as a need to focus efforts on those that were mission aligned amidst substantial competing priorities.

### Challenge 6: Representation, Representativeness, and Equity

Limitations in the representativeness of documented data or in the range of partners engaged can impede the ability to design equitable solutions. This may stem from difficulties in assessing equity of outcomes and having a limited number of partners to strategically address existing disparities [27,62,78]. Such limitations could exacerbate or create new inequities without the capability to use data to identify and resolve these issues. IS methods, including PRISM, promote representativeness in data and partner engagement, which may not always be achievable within existing constraints. Therefore, when planning LHS or learning cycles, equity should be clearly defined and prioritized from the outset [79].

To the extent feasible, inclusive use of data and engagement of partners across the spectrum of perspectives—not just the average or majority perspective—is important for promoting equity within LHS [27]. Proactively considering the potential unintended consequences of using different types of data is also key to mitigating inequities. When possible, integrating data sources beyond the EHR (eg, social media, patient and staff satisfaction, community forums, community partner data) and using systems science approaches that include patient-reported outcomes and other social determinants and behavioral data can aid in more comprehensive consideration of the data needed to promote health equity [80]. There is a clear need for health systems to access a more inclusive integration of reliable, structured data.

Across all examples, none were able to gain the breadth of partner perspectives that is ideal to sufficiently assess the representativeness of outcomes, but they did what was feasible. For instance, the representativeness (eg, gender, race, age) of clinicians who adopted the CDS or lung ultrasound was not assessed because these data are stored outside of the EHR and inaccessible to those evaluating this type of LHS work.

## Discussion

In many ways, EHRs have enabled the visionary idea of an LHS to become a reality for many health systems. Yet, as highlighted in our case studies, this reliance on EHRs also limits their potential. These case examples demonstrate how IS—when applied in practical, accessible, and adaptable ways—can help LHSs navigate the challenges posed by EHRs while also addressing other crucial factors, such as team science. We highlight aspects of EHR-based LHSs that can complicate the application of IS, notably limitations in the type and completeness of available data. To enable LHS to practically apply IS, our case studies illustrate how an IS framework and its methods (PRISM) can be adapted to drive meaningful change. While IS can sometimes appear overly academic, complex, or inflexible, we emphasize that IS should be tailored to fit specific situations. We encourage others to utilize existing tools and resources to make IS more accessible and practical for their needs.

A cross-cutting key take-home message for LHSs broadly, and particularly when applying IS to EHR-based LHS, is to “do what you can with what you have while proactively anticipating and mitigating unintended consequences and harm” [60,81]. Historically, health care has often pursued perfection, which has led to rigidity in applying IS methods and utilizing EHR data and technology. This mindset can significantly delay or impede progress and is at odds with the visionary goals of LHS, which emphasize practical, relevant, and rapid learning cycles. Perfection is neither realistic nor attainable, and while striving for a perfect solution, health systems, priorities, and innovations evolve quickly, rendering solutions obsolete before they are even implemented [82,83].

In our case studies, we also identified areas for future development to enhance the accessibility of IS and the utility of EHR data and technology for LHS. First, there is a need for more user-friendly tools and resources to guide the use and adaptation of IS TMFs and methods across various types of projects and situations. Such resources should provide guidance on simplifying the application and adaptation of TMFs, taking into account relevance, data, and resource constraints. They should also address designing for sustainability, equity, and generalizability, including for mandated projects that lack an evidence base. Additionally, these resources should help systematically anticipate and mitigate unanticipated consequences, including those that could potentially misinform future policy. Additionally, we reinforce the decade-long call for more inclusive integration of accessible, high-quality data essential for achieving precision health goals [84,85]. Change is needed to ensure that LHSs have equitable, collaborative, and agreed-upon access to a comprehensive range of data—such as mental, physical, and behavioral health information; social determinants; environmental risks; patient preferences; and genomic data—that drive equitable health care outcomes and are crucial for making informed, patient-centered, and personalized health care decisions [84,85].

Since the original call for LHS in 2007 [7], significant progress has been made, with a growing number of functional LHS [10,86]. EHRs provide foundational infrastructure and data that make LHS possible, and IS methods can help both new and existing LHSs achieve their goals of equitable, sustainable, reproducible, and relevant knowledge generation and translation. However, to foster the growth of new LHSs and support existing LHSs in achieving the aspirational goals of a fully mature, equitable, and sustainable LHS [10], there is a clear need for greater access to inclusive data and more guidance on the practical application of IS methods.

## Appendix

Multimedia Appendix 1 Examples of challenges and solutions to applying PRISM to EHR-based LHS research categorized according to whether the issue primarily stems from PRISM or the EHR.

Multimedia Appendix 2 Additional tools and resources for guidance on how to apply IS.

## Abbreviations

CDS: clinical decision support
EHR: electronic health record
FQHC: federally qualified health center
iPRISM: Iterative Practical, Robust Implementation and Sustainability Model
IS: implementation science
LHS: learning health system
PRISM: Practical, Robust Implementation and Sustainability Model
RE-AIM: Reach, Effectiveness, Adoption, Implementation, and Maintenance
TMF: theories, models, and frameworks
