# Supplementary material for: Applying Implementation Science to Advance Electronic Health Record–Driven Learning Health Systems: Case Studies, Challenges, and Recommendations
Source: J Med Internet Res. 2024 Oct 7;26:e55472. doi: 10.2196/55472 (PMC11494259; doi:10.2196/55472)
Supplement: Multimedia Appendix 1 [file jmir_v26i1e55472_app1.docx]

| **Appendix 1**. Examples of challenges and solutions to applying PRISM to EHR-based LHS research categorized according to whether the issue primarily stems from PRISM or the EHR | | | |
| --- | --- | --- | --- |
|  | **Challenges of applying PRISM and relying on EHR-focused data** | **How challenges were or could be addressed (solutions)** | **Future directions (unresolved challenges)** |
| EHR-embedded CDS for heart failure | **PRISM issues:**  Original definition of adoption measure was not applicable  Unable to measure implementation costs within resource constraints  Time required to ensure representation of perspectives and design the CDS tool delayed timeliness of implementing the CDS tool  **EHR issues:**  Data needed not available, not structured, or unreliable which limited ability to evaluate context, measure RE-AIM outcomes, or optimally build/deploy the CDS tool:   - Patient symptoms or history of intolerance is inconsistently documented and when documented it is in unstructured fields which limits the specificity and accuracy of CDS tools - Medication data is collected in a way that makes estimations of daily doses unreliable which limits the capability of CDS to close gaps in suboptimal target doses - Attribution of clinicians to a clinic that they practice at is not reliable and not documented when they practice at more than one clinic which limits pragmatic study designs (e.g., intervention allocation in cluster randomization) - Data available to characterize clinicians was limited to specialty, practice location and patient visit characteristics which was often inaccurate (e.g., incorrect assignment of specialty) - Limited, incomplete or conflicting data available to characterize patients based on social determinants of health - Technical constraints of the CDS software limited ability to tailor CDS to better align with the clinician’s workflow and reason for the patient visit | **PRISM issues:**  The original definition of adoption was modified to provide valuable information in a way that was relevant for the situation  For future CDS tool projects, we drafted a time-driven activity based cost template and action plan to estimate implementation time  For future CDS tool projects, we are adopting a mindset of creating a minimally viable product and not trying to develop a perfect CDS tool to increase timeliness of implementation and minimize resources  **EHR issues:**  Reported reach with non-specific denominator that included anyone with the diagnosis of heart failure  CDS tools did not conditionally alert based on patient symptoms and relied on clinicians to consider these issues (e.g., good enough)  The CDS tool was designed for new starts and did not make recommendations to titrate doses (e.g., good enough)  We evaluated the distribution of clinician visits across clinics and determined cross contamination would be minimal. This approach could be used to guide intervention allocation but does not control for changes practice sites over time.  We reported the data available and noted the limitations of using EHR data  Future projects could report available data along with the proportion that is missing  We designed the CDS tool to alert at the same time during every visit irrespective of varying clinician workflows and it would alert for all visits irrespective of the reason | **PRISM issues:**  More guidance on how/when to adapt PRISM and its RE-AIM measures for diverse projects  More guidance on how to qualitatively assess context to overcome limitations of quantitative EHR data (e.g., lack of data on clinician characteristics and patient social determinants)  Resources, tools and templates to provide general guidance on how to apply PRISM and in ways that flex based on resource and time constraints  **EHR issues:**  More inclusive integration and standardized structured documentation of data in EHRs, including data on:   - clinician characteristics (e.g., race, gender, ethnicity) - patient-reported outcomes and social determinants of health, including indices of social deprivation and data from the US census - prescription fill/dispense data - medication data captured in a way that facilitates identification of active medications and total daily doses   Greater ease of access to software and analytic skill (e.g., natural language processing, deep learning) to:   - transform unstructured free text into structured and actionable formats, including data on patient symptoms - analyze qualitative data   Expanded technical capabilities of CDS tool software to allow greater flexibility to align with clinician workflows and improve accuracy and specificity |
| **EHR-based dashboard for Lung Ultrasound Screening** | **PRISM issues:**  Not all RE-AIM outcomes were able to be assessed within resource constraints. For instance, fidelity assessment of the quality of imaging interpretation required chart review by an expert and took faculty time.  Reporting reach as just a percentage would be misleading because the number of patients eligible for lung ultrasound month to month varied throughout the 1-year pilot  Unable to measure implementation costs within resource constraints  Contextual assessments required qualitative interviews which took significant time and resources to conduct and analyze  Was not able to proactively screen for unintended consequences given resource constraints  **EHR issues:**  Not all data was discretely documented in the EHR which limited the ability to measure some RE-AIM outcomes like implementation, including fidelity, which required manual review by an expert. Some outcomes were not prioritized to be assessed given resource constraints.  Limited, incomplete or conflicting data available to characterize representativeness | **PRISM issues:**  In the future, could use more advanced analysis methods to capture and display RE-AIM outcomes in the dashboard:   - natural language processing (NLP) to capture unstructured data needed to measure certain outcomes - deep learning to assess the quality of images - NLP to assess quality of image interpretation and clinical decision-making   We reported reach in absolute numbers for increased transparency  In the future, could develop a time-driven activity based micro-costing approach that considered factors such as EHR time stamps or compare cost of outcomes between traditional and POCUS  In the future could use rapid qualitative methods in combination with advanced analysis methods (e.g., NLP)  In the future could assess for unintended consequences during qualitative interviews, by direct observation, manual chart review, or Use Operational Dashboard to visualize how other interventions or aspects of context are impacted by implementation of lung ultrasound  **EHR issues:**  In the future, could leverage NLP or deep learning to capture this unstructured data  Although limited, used data available including patient demographics (e.g., good enough) | **PRISM issues:**  More guidance on how to feasibly and systematically anticipate, mitigate and assess for unintended consequences including exacerbation of inequities when using PRISM  **EHR issues:**  Improve the standardization and structured documentation of clinical data and ease of access to advanced analytics such as NLP to increase access to data and enable better insights into contextual influences  More inclusive integration of data in EHRs, including:   - representativeness data - patient performed questionnaires and other patient-reported outcomes - Social determinants of health   Greater access to software and analytic skill (e.g., NLP, deep learning) to:   - transform unstructured free text into structured and actionable formats - analyze qualitative data and images |
| **FQHC-led social needs screening and response equity study** | **PRISM issues:**  PRISM was new to most partners and there was a need to develop a shared vocabulary and goals across the diverse perspectives of the implementation team.  PRISM and IS methods were new to some who had varying training and/or engagement. FQHCs often have numerous complex mandated quality improvement requirements that could benefit from IS methods, yet FQHC budgets and traditional staffing models frequently have limited expertise  Despite discussion that appeared to reach consensus, it was realized that each partner initially made sense of the process, overall goals and focus of the project differently, which resulted in partners completing the iPRISM webtool with a different interpretation of the project scope  Understanding the complex context and different partner perspectives can be time and resources intensive which:   - resulted in one partner being unable to attend one of the debrief meetings - limited representativeness of partners engaged including community members and patients   **EHR issues:**  Each FQHC has variable: EHR workflows, technical resources available to modify the EHR, and staff available to support new data collection approaches.  Many patient reported variables documented in the EHR (e.g., social risks and race, ethnicity, language), are complex to collect and ensure equity, self-reported accuracy and completeness. | **PRISM issues:**  Use of the iPRISM webtool early and iteratively in the planning phase helped define and document vocabulary and goals and created a shared learning environment where differences in role, scope, focus, and situational perspective were surfaced  Collaboration with methodologic experts and use of the iPRISM webtool guided partners through the process of systematically and consistently applying IS to assess PRISM’s context and anticipated RE-AIM outcomes. The embedded education within the iPRISM webtool provided guidance on developing meaningful outcomes measures and identifying important contextual issues  In the future, will anticipate the alternate perspectives and potential impact on each partner’s approach to completing the iPRISM webtool. Additional preparatory time will be spent to address and discuss this, ideally before applying the iPRISM webtool to the project, but also throughout the use of the iterative iPRISM webtool process.  All perspectives were considered via asynchronous responses to the iPRISM webtool and other strategies (e.g., shared meeting notes, emails to gain consensus and all perspectives).  In the future will try to engage additional partner perspectives including community and patients  **EHR issues:**  Each site used two population health tools that facilitated discrete documentation of screens in the EHR and automated data extraction and reporting across common data elements despite different EHRs. | **PRISM issues:**  Need for more guidance on how to design for sustainability and the future state, including identifying and securing resources to support the ongoing partnership efforts and related initiatives.  More guidance is needed on how to implement interventions when mandated without evidence of effectiveness  **EHR issues:**  Need more integration or interoperability of EHRs, data analytics, and population health tools to facilitate ease of LHS across diverse health settings and different EHRs |
