# Supplementary material for: Applying Implementation Science to Advance Electronic Health Record–Driven Learning Health Systems: Case Studies, Challenges, and Recommendations
Source: J Med Internet Res. 2024 Oct 7;26:e55472. doi: 10.2196/55472 (PMC11494259; doi:10.2196/55472)
Supplement: Multimedia Appendix 2 [file jmir_v26i1e55472_app2.docx]

| **Appendix 2. Additional tools and resources for guidance on how to apply IS** | |
| --- | --- |
| **Resource** | **Description** |
| iPRISM webtool  *(https://prismtool.org)* | An interactive website that guides and prompts individuals and teams through the process of operationalizing PRISM to assess context, align with the context, identify strategies and adaptations that are feasible and impactful, and develop action plans. Can be used by English or Spanish speaking teams or individuals and provides useful visual feedback displays that can be used to identify areas in need of improvement. |
| A Guidebook to the Pragmatic and Iterative Use of the PRISM and RE-AIM for Planning, Implementation, and Sustainment  *(https://medschool.cuanschutz.edu/accords/cores-and-programs/dissemination-implementation-science-program/resources-services#Resources-Services-InteractiveTools)* | A comprehensive and detailed step-by-step summary of different ways to apply PRISM, including using it iteratively and with teams of implementers; for different project phases; and with or without RE-AIM outcomes. Includes example PRISM surveys and templates. |
| RE-AIM website  *(https://RE-AIM.org)* | An online and frequently updated repository of references, resources, tutorials, videos and examples related to RE-AIM and PRISM. |
| CFIR website (Consolidated Framework for Implementation Research)  (*https://cfirguide.org/)* | An online and frequently updated repository of references and resources related to CFIR. |
| Dissemination & Implementation Models in Health  (*https://dissemination-implementation.org/)* | An online and frequently updated searchable repository of theories, models and frameworks that allows user to query by various characteristics such as stage of implementation. |
| Mi-PARIHS Facilitation Planning Tool  (*https://www.flinders.edu.au/caring-futures-institute/do/mi-parihs)* | A resource to develop a facilitation plan using the i-PARIHS (integrated-Promoting Action on Research Implementation in Health Services) framework. The tool provides guidance for working through the i-PARIHS framework to identify key barriers and enablers and develop a facilitation plan tailored to the recipients and context. |
| The Hexagon tool  (*https://implementation.fpg.unc.edu/resource/the-hexagon-an-exploration-tool/)* | A tool intended to be used by a team to evaluate both new and existing programs and practices. It assists the team in comparing program indicators (usability, evidence, supports) with implementing site indicators (fit, need, capacity) to assess whether the new program or practice is a strong match with the implementation site. |
| Program (PSAT) and  Clinical Sustainability Assessment Tools (CSAT)  *(https://www.sustaintool.org/psat/*) | Online interactive tools to guide evaluation of sustainability. The PSAT is for community and public health programs and the CSAT for clinical interventions. |
| DICEMethods webtool  (*https://dicemethods.org/)* | An interactive online tool that offers guidance on partner engagement methods for clinical and translational projects. Users are able to answer a few questions related to their project including research stage, purpose, and budget and the tool will recommend specific methods. |
| National Cancer Institute Implementation Science Research and Practice Tools  *(https://cancercontrol.cancer.gov/is/tools/research-tools)* | An online repository of tools and resources to help researchers and practitioners understand, plan for and implement projects using dissemination and implementation science methods, including: theories, frameworks, and models; measures; strategies; and research methods and study design. The website includes archived webinars and research articles for these different topics. |
